# Involvement of Kallikrein-Related Peptidases in Nervous System Disorders

**DOI:** 10.3389/fncel.2020.00166

**Published:** 2020-06-23

**Authors:** Cinthia Mella, Carlos D. Figueroa, Carola Otth, Pamela Ehrenfeld

**Affiliations:** ^1^Faculty of Medicine, Institute of Clinical Microbiology, Universidad Austral de Chile, Valdivia, Chile; ^2^Laboratory of Cellular Pathology, Institute of Anatomy, Histology, and Pathology, Universidad Austral de Chile, Valdivia, Chile; ^3^Center for Interdisciplinary Studies on the Nervous System (CISNe), Universidad Austral de Chile, Valdivia, Chile

**Keywords:** KLK6, KLK8, central nervous system, viral infection, kallikrein-related peptidases

## Abstract

Kallikrein-related peptidases (KLKs) are a family of serine proteases that when dysregulated may contribute to neuroinflammation and neurodegeneration. In the present review article, we describe what is known about their physiological and pathological roles with an emphasis on KLK6 and KLK8, two KLKs that are highly expressed in the adult central nervous system (CNS). Altered expression and activity of KLK6 have been linked to brain physiology and the development of multiple sclerosis. On the other hand, altered levels of KLK6 in the brain and serum of people affected by Alzheimer’s disease and Parkinson’s disease have been documented, pointing out to its function in amyloid metabolism and development of synucleinopathies. People who have structural genetic variants of KLK8 can suffer mental illnesses such as intellectual and learning disabilities, seizures, and autism. Increased expression of KLK8 has also been implicated in schizophrenia, bipolar disorder, and depression. Also, we discuss the possible link that exists between KLKs activity and certain viral infections that can affect the nervous system. Although little is known about the exact mechanisms that mediate KLKs function and their participation in neuroinflammatory and neurodegenerative disorders will open a new field to develop novel therapies to modulate their levels and/or activity and their harmful effects on the CNS.

## Introduction

**Figure 1:**
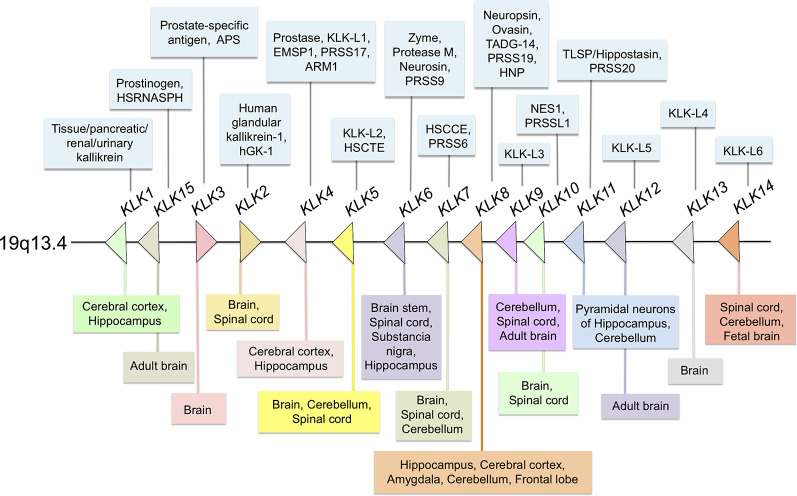
Distribution of all members of the human kallikrein family at different sites of the central nervous system (CNS). Most members of the family are expressed in the human or rodent CNSs. In the center (arrows) Kallikreins genes distribution in 19q13.4 chromosome; in the upper section (light blue boxes) others, protein or gene names/symbols for each KLK; in the lower panel (multi-colored boxes) reported localization in the CNS and spinal cord.

The kallikrein-related peptidases are a family of trypsin- and chymotrypsin-like serine proteases, which have significant differences regarding their amino acid sequence, relative molecular mass, substrate specificity and also, biochemical and functional properties (Diamandis and Yousef, 2001; Yousef and Diamandis, 2001; Stefanini et al., 2015). Originally, they were designated *kallikrein-like-kallikreins* (KLKs) because of their proximity in the chromosome to the *KLK1* gene that encodes true kallikrein, the primary kinin-releasing enzyme in humans, discovered in the pancreas (Bhoola et al., 1992). Like KLK1, the other human KLKs (KLK2–KLK15) belong to a highly homologous serine protease family whose genes are located in tandem on chromosome 19q13.4 (see Figure 1) and comprise the largest group of genes encoding proteases in the human genome. In the present review article, we examine the current knowledge on the pathological implications and normal functions of human KLKs in the central nervous system (CNS) with special emphasis on KLK6 and KLK8, two members of the family that are highly expressed in the adult brain and participate in processes such as synaptic plasticity and brain development in normal CNS.

## KLKs as Signaling Molecules

KLKs are secreted as pro-enzymes, for which they require the removal of their terminal peptide through specific amino-terminal proteolysis. Some KLKs activate other pro-KLKs *in vitro*, while others have the ability of autocleavage, and some suffer cleavage by other proteases, such as metalloproteases (MMPs). Therefore it is suggested that they participate in cascade-like enzymatic pathways (Davie et al., 1991; Pampalakis and Sotiropoulou, 2007; Sotiropoulou et al., 2009). Through their enzymatic activity, KLKs regulate essential physiological functions, such as immunity (Shaw et al., 2008; Ehrenfeld et al., 2018), skin desquamation (Brattsand et al., 2005) enamel formation (Simmer et al., 1998; Wright et al., 2006), and semen liquefaction (Emami et al., 2008). Also, KLKs have been shown to modulate the bioavailability of growth factors, to activate protease-activated receptors (PARs), increase cell proliferation, and degrade different components of the extracellular matrix (ECM). In this respect, Iwadate et al. (2003) showed that some neurons express KLK1 and Insulin-like growth factor-binding protein-5 (IGFBP-5); KLK1 cleaved IGFBP-5 at two different points suggesting a role in brain physiology by regulating the availability of IGFBP-5 and IGF to neurons. Likewise, KLK6 can release enkephalin from pro-enkephalin precursors, similarly to the canonical processing performed by the enzyme furin (Silva et al., 2017). It has been proposed that alterations in the enzymatic pathway that controls the maturation of pro-nerve growth factor (NGF) to NGF and its subsequent degradation could be relevant in neurodegenerative processes such as Alzheimer’s disease that affect the activity of basal cholinergic neurons of the forebrain (Fahnestock and Shekari, 2019; Mitra et al., 2019; Mufson et al., 2019). A role for KLK8 in the expression of NGF at the skin has been described, but studies directed to show whether that KLK8 or other KLKs influence the bioavailability of NGF in the CNS have not yet been explored (Shingaki et al., 2012).

The evidence supports that KLKs are also involved in tumorigenesis by activating proteolytic processes, which are relevant to neoplasia. Indeed, KLKs promote the migration and invasion of tumor cells (e.g., malignant breast tumors that spread into the brain) by favoring the epithelial-mesenchymal transition (EMT; Witzel et al., 2016; Brosnan and Anders, 2018; Franchino et al., 2018; Takahashi and Isogawa, 2018). KLKs have also been suggested as modulators of the interactions occurring between the cells that shape tumor microenvironment, to promote angiogenesis and other pro-tumorigenic processes (Stefanini et al., 2015; Filippou et al., 2016; Kryza et al., 2016).

Tissue-specific deregulation of KLKs has been associated with different disorders, including respiratory diseases, schizophrenia, neurodegeneration, anxiety, defective cutaneous barrier, inflammation, and cancer, among others (Stefanini et al., 2015). Endogenous regulation of KLKs is controlled at different levels (Goettig et al., 2010), which constitute potential therapeutic targets to control their dysregulation in different types of disorders (Vandell et al., 2008; Kalinska et al., 2016). Research on the different members of the kallikrein family has become important since nowadays it is known that an increased or decreased expression of a particular KLK may be an indicator for certain neurodegenerative diseases. For this reason, some KLKs have been proposed as biomarkers for particular disorders of the CNS.

### KLKs and Protease-Activated Receptors (PARs)

**Figure 2:**
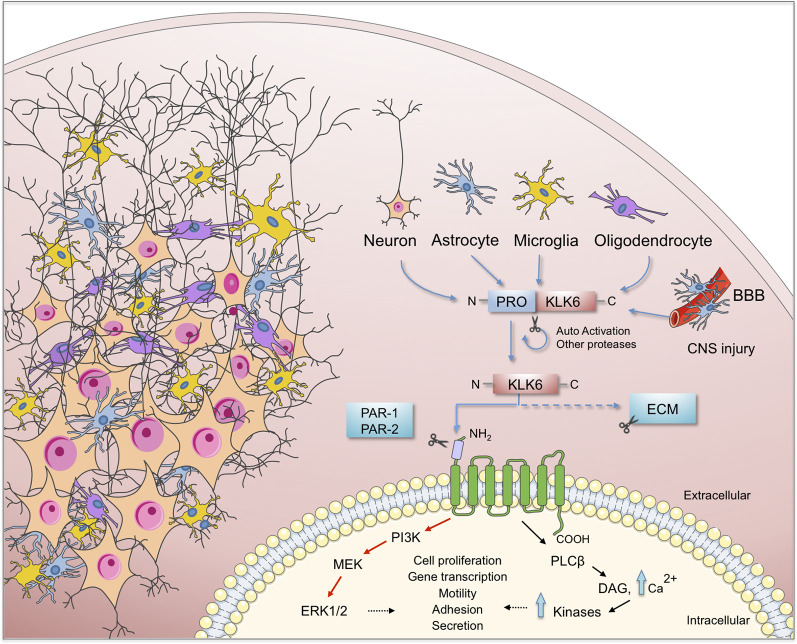
Schematic representation of the KLK6 self-regulation and the major effects of it functions on brain physiology. KLK6 is released by glial and neuronal cells to cleave ECM components and proteinase-activated receptors (PARs) which are important steps in the inflammatory response during the pathogenesis of neurodegenerative diseases. ECM, extracellular matrix; BBB, blood-brain barrier; CNS, central nervous system.

As proteases, KLKs trigger intracellular signaling pathways by activating PARs, G protein-coupled receptors located in the cell membrane of many cells throughout the body. In the nervous system PARs can be found in neurons (Striggow et al., 2002), microglia (Suo et al., 2003), astrocytes (Wang et al., 2002) and oligodendrocytes (Wang et al., 2004). Meanwhile, 13 KLKs are expressed in the human brain (Figure 1) of which 10 have been considered as PAR activators. Additionally, Borgoño et al. (2007) reported that KLK3, KLK7, and KLK9 are chymotrypsin-like proteases, and therefore it is unlikely that they cling to PARs. Following PAR activation by KLKs, downstream signaling pathways that regulate different physiological functions are activated. Among them is the pathway of mitogen-activated protein kinases (MAPKs) that link PARs with cellular proliferative responses and the nuclear factor-kappa B (NF-κB) pathway that modulates proinflammatory responses (Oikonomopoulou et al., 2006; Rothmeier and Ruf, 2012; see Figure 2). PAR-1 agonists cause a rapid and transient contraction of endothelial cells in various tissues, leading to a hyperpermeable vascular wall, which allows the leakage of plasma proteins and may favor the migration of inflammatory cells; activation of PAR-2 also leads to an increase in vascular permeability, leukocyte margination and extravasation (Vergnolle, 1999). All these events may contribute to developing an acute inflammatory environment through PAR-1, increasing the production of IL-1, TNF-α, IL-6, MCP-1, and IL-10, but down-regulating IL-12 secretion; similarly, PAR-2 signaling increases the production of IL-1, IL-6 and IL-8 (Chen and Dorling, 2009) contributing to neuroinflammation when the blood-brain barrier (BBB) is disrupted by an injury affecting the CNS. KLKs are important PAR activators in the CNS being KLK6 one of the more important partners under both physiological and pathological circumstances (see below).

## KLKs in The Central Nervous System

It is known that all kallikreins are expressed at varying levels in the CNS. Interestingly, KLK3 mRNA transcripts were detected by RT-PCR and KLK3 protein was visualized in neuronal cells by immunohistochemistry (Stone et al., 2009). By comparison, low levels of KLK15 mRNA and protein were identified in human brain tissue extracts by using RT-qPCR and ELISA, respectively (Shaw and Diamandis, 2007). On the other hand, *KLK6* to *KLK12* and *KLK14* mRNAs are expressed in relatively larger amounts than the other KLKs (Yousef et al., 2003). At the protein level, the presence of seven KLKs was confirmed in protein extracts of the adult brain (Shaw and Diamandis, 2007). Furthermore, KLK13 was found in neurons and glial cells by using immunohistochemistry (Petraki et al., 2003). A comparison between mRNA levels in the brain and those expressed in the spinal cord indicates that *KLK6*, *KLK8*, and *KLK10* are expressed at significantly higher levels in the spinal cord. By contrast, *KLKs* *1*, *2*, *5*, *7*, *9*, *12*, and *14* are more abundant in the brain, with *KLK*6 levels reaching approximately 10-fold more than other members of the family (Scarisbrick et al., 2006a). A summarized distribution of the KLK family in the CNS is shown in Figure 1. For a comprehensive report on the physiology and pathophysiology of KLKs in several disorders of the CNS see Scarisbrick (2012).

Extracellular serine proteases play essential roles in the normal physiology of the nervous system, including neurite maturation, synaptic plasticity, and neuronal degeneration (Xi et al., 2003). Among them, KLKs have been implicated in the pathogenesis of neurodegenerative diseases such as Alzheimer’s disease (Diamandis et al., 2000; Shimizu-Okabe et al., 2001; Ashby et al., 2010; Herring et al., 2016), Parkinson’s disease (Ogawa et al., 2000; Iwata et al., 2003) and multiple sclerosis (Scarisbrick et al., 2002; Panos et al., 2014; Yoon and Scarisbrick, 2016).

To date, several reports have shown that of all KLKs at least two murine kallikreins, Klk6 (Klk, nomenclature for a particular KLK in the mouse and rat) and Klk8 are associated with important processes such as synaptic plasticity and brain function (Little et al., 1997; Scarisbrick et al., 1997). KLK6, the human counterpart of Klk6, is preferentially expressed in the CNS compared to non-neural tissues. KLK6 was first described in serum-free supernatants of a human colon adenocarcinoma cell line (Yamashiro et al., 1997). KLK6 is down-regulated in breast cancer cell lines characterized by a high degree of malignancy but is strongly expressed at the mRNA level in primary cultures of breast cancer cells and ovarian cancer tissue and ovarian cancer cell lines (Anisowicz et al., 1996).

## Bioregulation of KLK6 and KLK8 in Normal and Pathological Conditions of The CNS

### KLK6

Human KLK6 is a trypsin-like serine protease that can degrade various components of the ECM, including fibronectin, fibrinogen, laminin, vitronectin, and collagen (Yousef and Diamandis, 2001; Ghosh et al., 2004). Some of these ECM molecules have been involved in synaptic maturation and plasticity in the brain (Kwok et al., 2011). KLK6 is abundantly expressed in the peripheral nervous system and the CNS (Scarisbrick et al., 1997), is mostly secreted by oligodendrocytes, pyramidal cells and astrocytes (Prassas et al., 2015). Although KLK6 is not expressed in astrocytes or microglia during their resting state, it is expressed by each of these cell types in response to injury (Scarisbrick et al., 2006b, 2012a; see Figure 2).

KLK6 has been involved in the regulation of myelin sheath volume (Blaber et al., 2002) and degradation of a β-amyloid peptide, a function associated to different neurodegenerative diseases such as Alzheimer’s disease, in which it has been proposed as a biomarker (Diamandis et al., 2000). There is a decrease of KLK6 protein in the serum of patients with Alzheimer’s disease compared to control patients of the same age (Menendez-Gonzalez et al., 2008). Furthermore, the brain of Alzheimer’s disease patients contains significantly lower KLK6 levels than the brain of nonaffected individuals (Zarghooni et al., 2002; Ashby et al., 2010); when the mRNA levels were quantified in the hippocampus they were also decreased (Shimizu-Okabe et al., 2001). Nevertheless, it has recently been reported that KLK6 levels in cerebrospinal fluid (CSF) are significantly increased in patients with Alzheimer’s disease and elevated CSF phosphorylated tau (p-Tau), but not in Alzheimer’s disease patients with normal CSF p-Tau levels. This observation may indicate that KLK6 is associated with Tau pathology and has the potential to be a suitable biomarker in tau pathology Alzheimer’s disease-related (Goldhardt et al., 2019). For a summary of some physiological functions and association of KLK6 deregulation with the disease, see Table 1.

**Table 1:** Main studies on KLK6 deregulation in humans and rodents (Klk6).

Localization	Sample	Technique	Physiological effect	Disease	Reference
Macrophages, Microglia, and Astrocytes	Rat or Human Spinal cord	IHC	System development, plasticity, and response to injury	Spinal cord injury	Scarisbrick et al. (2006b)
Oligodendrocytes	Adult Rat spinal cord white matter	*In situ* hybridization Histochemistry	Oligodendrocyte and myelin biology	White matter pathology	Scarisbrick et al. (2000)
Neurons, Astrocytes	NSC34 neuron and Neu7 astrocyte cell lines, primary cultures of cortical neurons and astrocytes from C57/BL6J mice	RT-qPCR, Calcium imaging, Western blotting	Promotion of astrocyte survival and astrogliosis through PARs	CNS injury scarification	Vandell et al. (2008)
Spinal cord	Spinal cord and splenocyte mice cultures with experimental autoimmune encephalomyelitis	RT-qPCR, Western blotting	The multifunctional driver of MS pathogenesis through neuroinflammatory and neurodegenerative processes	Inflammation in MS	Yoon and Scarisbrick (2016)
Astrocytes	Human spinal cord from MS and Glioblastoma patients, Murine primary cultures of astrocytes and Neu7 cell line	IHC RT-qPCR ELISA	Endogenous regulator of astrocyte physiology	Reactive astrogliosis at sites of CNS pathology	Scarisbrick et al. (2012a)
Brain, spinal cord	Mice spinal cord and splenocyte cultures	IHC RT-qPCR Western blotting	Regulation of development and progression of inflammation and demyelination	Active MS lesions	Scarisbrick et al. (2012b)
Brain	Post-mortem brain of AD and vascular dementia patients	IHC RT-qPCR Western blotting	KLK6 may contribute to vascular abnormalities in AD and vascular dementia	Vascular abnormalities in AD and vascular dementia	Ashby et al. (2010)
Oligodendrocyte	Oligodendrocyte primary cultures from wild type or PAR-1 KO mice and Oli-neu cell line	IHC RT-qPCR Western blotting	Functional role in PAR-1 signaling in oligodendroglia	CNS white matter injury	Burda et al. (2013)
Brain and spinal cord	Brain, spinal cord and splenocyte cultures of mice with MS	RT-qPCR	inflammation, demyelination and progressive axon degeneration in MS	Inflammatory pathology in MS	Panos et al. (2014)
Neurons	Primary cultures of cortical neurons from wild type and Klk6 KO mice	Zymography Western blotting	KLK6 can readily cleave α-synuclein fibrils	PD and other synucleinopathies	Pampalakis et al. (2017)
CSF	CSF from AD patients	ELISA	CSF-KLK6 elevation association with Tau pathology in AD	Tau pathology in AD	Goldhardt et al. (2019)

*KLK6, Kallikrein-related peptidase 6; IHC, Immunohistochemistry; RT-qPCR, Quantitative reverse transcription Polymerase Chain Reaction; ELISA, Enzyme-linked immunosorbent assay; CNS, Central nervous system; KO, knockout; CSF, Cerebrospinal fluid; PAR, Protease-activated receptor; AD, Alzheimer’s disease; MS, Multiple Sclerosis; PD, Parkinson’s disease*.

Expression of KLK6 has also been reported in a wide range of neurons (Scarisbrick, 2012) and high protein and mRNA levels are expressed in the hippocampus (Scarisbrick et al., 1997, 2004). These authors also reported enriched expression of KLK6 protein in the frontal lobe, subthalamic nucleus, substantia nigra, thalamus, putamen, and caudate nucleus. By using immunohistochemistry, Petraki et al. (2001) reported KLK6 immunoreactivity in epithelial cells of choroid plexus, cerebellum, and Purkinje cells. Also, they reported KLK6 protein expression in the stellate (basket cells), glial cells, and peripheral nerves. Furthermore, the presence of KLK6 has also been described in the pituitary gland (Komatsu et al., 2007).

Evidence indicates that KLK6 is widely localized in the CNS, and its deregulation may be of relevance in a variety of neurological disorders. Thus, high levels of KLK6 have been found in actively demyelinating multiple sclerosis and spinal cord injury. Additional studies functionally link Klk6 to the onset and progression of multiple sclerosis (Scarisbrick et al., 2012b) since elevation in systemic KLK6 during the disease may lead the immune system towards a pro-inflammatory response that may exacerbate the disease by favoring neuroinflammation. This observation is supported by several findings showing abundant KLK6 expression in inflammatory cell subsets within the CNS. Leukocytes aggregate around the perivascular space and at sites of demyelination in animal models and individuals with multiple sclerosis (Scarisbrick et al., 2008, 2011; Panos et al., 2014). The asseveration that KLK6 participates in inflammatory CNS demyelination is supported by the fact that this protease exhibits digestive patterns against myelin basic protein (Bernett et al., 2002), which is responsible for adhesion of the cytosolic surfaces of multilayered compact myelin into myelin sheaths, necessary for the normal activity of the vertebrate nervous system. High levels of KLK6 may favor the progression of multiple sclerosis through an excessive cleavage of myelin basic protein, the most widely studied myelin protein in this disease. Interestingly, the capacity to cleave myelin basic protein has also been attributed to KLK13 (Andrade et al., 2011), but has not been related to the pathogenesis of multiple sclerosis.

During the curse of induced progressive demyelination, there is a positive regulation of Klk6 in the brain and the spinal cord, predominantly expressed by immune cells at sites of active demyelination in multiple sclerosis lesions (Yoon and Scarisbrick, 2016), demonstrating its contribution to inflammation, demyelination, and progressive axon degeneration (Panos et al., 2014). Interestingly, prior evidence demonstrated that Klk6 neutralizing antibodies attenuated the disease in autoimmune and viral murine models of the disease (Blaber et al., 2004; Scarisbrick et al., 2012b). Altogether, these findings suggest that overexpression of KLK6 worsens the outcome of the disease and may, therefore, serve as a useful therapeutic target for this disease.

As previously mentioned KLK6 can activate PAR-1 and PAR-2 in response to CNS damage, which leads to activation of several intracellular signaling pathways that may contribute to neurotoxicity (Smirnova et al., 1998, 2001; Citron et al., 2000; Vandell et al., 2008; Yoon et al., 2013). PAR activation by KLK6 triggers multiple signal transduction pathways in NSC34 neurons and Neu7 astrocytes (Vandell et al., 2008) suggesting that an important interrelationship may exist between both KLK6 and PARs *in vivo* to modulate relevant CNS physiologic functions. Performing microinjection of Klk6 protein into the dorsal column white matter of PAR-1 wild type and PAR-1 knockout mice, Burda et al. (2013) reported that PAR-1 promotes vacuolating myelopathy and loss of myelin basic protein, suggesting that KLK6 may regulate key biological and pathophysiological aspects of oligodendrocytes.

KLK6 can also cleave the N-terminal end of the kinin B2 receptor (B2R) triggering specific signaling pathways such as intracellular Ca^2+^ mobilization in NSC34 neurons and Neu7 astrocytes. The intracellular Ca^2+^ flux evoked by KLK6 is mediated by PAR-1 in neurons and both PAR-1 and PAR-2 in astrocytes (Vandell et al., 2008). Also, KLK6 modified the activation status of MAPKs and Akt signaling pathways. This protease promoted activation of Akt in astrocytes, but suppressed this signaling route in neurons, suggesting that activities such as cell differentiation and survival can be regulated in a particular way. Recently, Yoon et al. (2018) reported that Klk6 can influence astrocyte shape increasing intracellular Ca^2+^ following activation of PAR-1. Importantly, genetic deletion of PAR-1 reduced the neurotoxic effects produced by Klk6 (Yoon et al., 2013) indicating that under pathological circumstances the Klk6-PAR-1 axis may play an important role contributing to neurodegeneration. Thus, the use of PAR-1 antagonists or Klk6 selective inhibitors may arise as new therapeutic tools to reduce demyelination and favor myelin regeneration.

Although KLK6 can activate PAR-1, PAR-2, and kinin B2R, KLK1 only activates kinin B2R, being unable to activate PARs. This evidence points out new functions for KLKs in the specific regulation of signal transduction pathways that may be critical for some physiological activities of the CNS, such as cell differentiation and survival (Vandell et al., 2008). KLK6 can be found in its free form or as a complex with the endogenous protease inhibitor, alpha-1-antitrypsin (Korbakis et al., 2017). On the other hand, alpha-1-antitrypsin is known for its anti-apoptotic and anti-inflammatory properties, and it has been proposed that it could inhibit the NLRP3-inflammasome (NOD type family of receptors, pyrin domain containing 3) and neuroinflammation. Ebrahimi et al. (2018) linked this information to the neuronal protection exhibited by alpha-1-antitrypsin against glutamate-induced toxicity and the reduction of inflammation induced by Amyloid β1–42 (Aβ1–42) in microglial cells during Alzheimer’s disease. Moreover, it has been shown that stimulation with Aβ1–42 positively regulates NLRP3, caspase 1, and its astrocyte cleavage products. Furthermore, alpha-1-antitrypsin hinders these neuroinflammatory effects in a time-dependent manner suggesting that it may be a pharmacological target for Alzheimer’s disease treatment. Whether KLK6 negatively regulates alpha-1-antitrypsin and NLRP3 in the nervous system and dysregulation in KLK6 levels can affect the modulation of alpha-1-antitrypsin bioavailability and neuroinflammation is still an unexplored subject.

One of the histopathological hallmarks of Parkinson’s disease is protein aggregates called Lewy bodies and Lewy neurites, protein aggregations composed mostly of the protein α-synuclein. Current evidence indicates that extracellular aggregation of α-synuclein may be implicated in the pathogenesis of Parkinson’s disease and that KLK6 mediates its degradation directly and *via* a proteolytic cascade that involves MMPs (Pampalakis et al., 2017). Cleavage of α-synuclein seems to generate proteolytic fragments that inhibit the aggregation of intracellular α-synuclein (Iwata et al., 2003). Abnormal α-synuclein can disseminate to neighboring brain areas and be later internalized into the intracellular compartment in a manner that resembles prion diseases; according to a “prion-like” hypothesis, abnormal α-synuclein might accelerate Parkinson’s disease (Ma et al., 2019). α-synuclein lacks a signal peptide, but it is released into the ECM to reach later the plasma and CSF. Of the human proteases, MMP-1, -2, -9, and -14 (Sung et al., 2005), plasmin, and KLK6 have been shown to cleave extracellular α-synuclein *in vitro*. KLK6 is one of the most abundant serine proteases in the CSF and its levels are reduced in patients with synucleinopathy, including Parkinson’s disease (Miners et al., 2014). Now, Pampalakis et al. (2017) have identified pro-MMP-2 as a protease-activated by KLK6 that cleaves α-synuclein to produce potential fragments for the cell to cell propagation in Parkinson’s disease.

These findings suggest that KLK6 is an important protease implicated in different CNS disorders in which its expression is altered in a disease-dependent manner, indicating that KLK6 may be an important therapeutic candidate to be used to ameliorate some of the effects produced by multiple sclerosis or Parkinson’s disease.

### KLK8

KLK8 is expressed in the hippocampus, the lateral nucleus of the amygdala, and in other areas of the limbic system, all of which are associated with learning and memory (Ishikawa et al., 2011; Shiosaka and Ishikawa, 2011). A detailed study on this protease’s structural determinants of specificity, regulation of its activity, and substrate profiles can be found in Debela et al. (2018).

Regarding its involvement in memory, KLK8 is activated through a mechanism that is linked with NMDA receptors (N-methyl-d-aspartate) and dual-specific mitogen-activated kinase 1 (MEK1). Interestingly, an early study proposed that activated KLK8 directly and specifically modifies the synaptic adhesion molecule L1 (L1CAM or NCAML1) by cleaving it in the pre-synaptic zone, releasing a neuropsin-specific extracellular 180 kDa fragment involved in NMDA receptor-dependent synaptic plasticity, which results in improved Schaffer collateral LTP (Matsumoto-Miyai et al., 2003). The *in vitro* results obtained by Tamura et al. (2012) suggest that KLK8 controls gamma-aminobutyric acid (GABA) neurotransmission using neuregulin-1 (NRG-1) and its receptor, ErbB4, modulating neural plasticity. KLK8 is significantly involved in the development of hippocampal memory dependent formation (Tamura et al., 2006; Suzuki et al., 2014), demonstrating that this protease has an important role in CSN physiology, particularly in neural plasticity and memory. However, the precise KLK8 signaling that confers its role in memory is still not clear.

Studies performed on Klk8-deficient mice showed that they present drastic deterioration of early-phase LTP in the Schaffer collateral pathway and hippocampal-dependent memory. Therefore, it is suggested that extracellular proteolysis contributes to LTP by modifying the adhesive relations of synapses (Ishikawa et al., 2008, 2011; Shiosaka and Ishikawa, 2011). In the brain, expression of KLK8 was observed in neurons and white matter axons, after experimental spinal cord injury and human traumatic spinal cord damage (Radulovic et al., 2013). Also, Panos et al. (2014) reported a robust upregulation of KLK8 during the acute phase of viral encephalitis in an experimental model of multiple sclerosis, in the brain and the spinal cord during development and progression of the disease. These findings point out an important role for KLK8 in inflammation, demyelination and progressive axon degeneration during disease.

In this context, several reports have evaluated the cerebral expression of Klk8 at both mRNA and protein levels during Alzheimer’s disease; the impact of Klk8 inhibition on Alzheimer’s disease-related pathology in mice, and primary glial cells has also been tested. At initial stages of the disease, Klk8 mRNA and protein are up-regulated, whereas KLK8 inhibition disrupts the processing of amyloid precursor protein, increases autophagy, reduces Aβ load, and Tau pathology, facilitates the elimination of Aβ through the BBB, improving neuroplasticity and memory, and reduces anxiety and fear. These observations suggest that inhibition of KLK8 may be considered as a key target during the treatment of Alzheimer’s disease (Herring et al., 2016). KLK8 may regulate the expression of microtubule-associated protein 2 (MAP-2), dendrite architecture, and protein kinase A (PKA)-CREB signaling, which indicates that KLK8 is an essential regulator of memory (Konar et al., 2018). Also, the influence of gender, specifically female, in the incidence of developing Alzheimer’s disease and the progression of various aspects of the disease, was recently reported using transgenic CRND8 mice. According to this investigation, Klk8 levels are higher in females than in male brains during the early stages of the disease when no plaques are detectable. This might be due to sex-differences that emerged after the onset of Alzheimer’s disease and because estradiol, but not testosterone induces Klk8 synthesis in neurons and microglia. After quantifying cerebral KLK8 levels in Alzheimer’s disease patients they found that its levels were similar to those described in mice. Moreover, high KLK8 levels were recorded not only in Alzheimer’s disease-affected subjects but also in the healthy brains of women. This evidence suggests that KLK8 overexpression might be an important factor for the preferential prevalence of Alzheimer’s disease in females (Keyvani et al., 2018). At date, it remains to be clarified whether KLK8 requires to trigger a PAR signaling route to exercise its activities in the nervous system.

Individuals carrying structural genetic variants encompassing *KLK8*, manifest mental illness such as intellectual and learning disabilities, seizures, and autism (Firth et al., 2009). KLK8 has also been implicated in schizophrenia, bipolar disorder (Izumi et al., 2008) and depression, in which patients with recurrent depression manifest higher *KLK8* expression than first-episode patients (Talarowska et al., 2016; Bobińska et al., 2017; Starnawska et al., 2019).

Interestingly, thrombin, a serine protease, involved in the coagulation cascade, has been shown to affect neuronal function following BBB breakdown. In the CNS different concentrations of thrombin affect LTP in mice hippocampal neurons through different molecular routes converging on PAR-1 (Maggio et al., 2013). By activating PAR-1, PAR-3, and PAR-4, thrombin can exert a wide variety of effects including vasodilatation, increased vascular permeability, and chemotaxis (Vergnolle, 2000; Chen and Dorling, 2009). A possible interrelationship between KLK8 and thrombin in the LTP in hippocampal neurons through PAR-1 has not been explored yet. Besides all the information referring to KLK6 and KLK8, such as their aberrant expression in Alzheimer’s disease and dementia (Ashby et al., 2010), a recent study expanded the KLKs contribution in CNS disorders by showing that the brain of Alzheimer’s disease patients presents decreased KLK7 mRNA expression. Moreover, KLK7 ablation exacerbated amyloid deposits in a mouse model of Alzheimer’s disease supporting the idea that KLK7 is a major peptidase involved in the degradation and clearance of deposited Aβ species (Kidana et al., 2018).

## KLKs Associated to Viral Infections That Can Affect The CNS

Despite having a significant role in different physiological processes, KLKs have also been attributed to a role in infectious processes such as those mediated by viruses, some of them related to neurological disorders. Recent studies have shown the importance of KLK8 during viral infections, such as those produced by the human papillomavirus (HPV). It has been proposed that KLK8 is involved in the entry of the virus into the cells through the proteolytic cleavage of L1 protein, the main capsid protein of HPV (Cerqueira et al., 2015; Becker et al., 2018). Remarkably, viral agents like HPV are believed to participate in the pathogenesis of multiple sclerosis where viral components act as a short-term risk factor for multiple sclerosis onset or relapse in the established disease. However, this hypothesis is still controversial considering the low incidence of multiple sclerosis in young people (Aghaei et al., 2017; Meggiolaro et al., 2018). HPV infections have also been related to gliomagenesis and the occurrence of glioblastoma multiforme (Vidone et al., 2014). Interestingly, at the date, the possible role or differential regulation/function of KLK8 and/or KLK6 in multiple sclerosis or other neural diseases associated with viral infections remains to be elucidated.

It has also been demonstrated that KLK5 and KLK12 are involved in the proteolysis that causes the conformational change of the hemagglutinin protein, which allows the fusion of influenza A virus (IAV) with the host endosomes, favoring the release of the viral genome. Curiously, knowledge of the interactions that may occur between IAV and the CNS cells is still limited, and therefore the studies of viral factors that are important for efficient replication in the CNS are scarce. Despite that, it was recently elucidated that a special hemagglutinin cleavage mechanism and preference for α-2, 3 linked sialic acids are necessary to allow virus attachment, contributing to the ability of IAV to efficiently infect neuronal cells in the CNS (Yao et al., 2008). This virus usually infects the cells of the upper and lower respiratory tract in humans where the infection begins with the attachment of viral proteins to sialic acid residues in the host cell. Little is known about sialic acid cell distribution, but a lectin immunohistochemical study performed in humans suggested that in different regions of the brain (brainstem, hippocampus, cerebellum, and cerebral cortex), both neurons and glial cells have α-2, 6 and α-2, 3 sialic acid residues (Siegers et al., 2019). If KLK5, KLK12, or others contribute to modulate the sialic acid cell distribution for the entrance of viruses into the CNS remains to be elucidated.

CNS infection is one of the most common extra-respiratory tract complications of IAV transmission. Importantly, differences among seasonal, pandemic, and zoonotic influenza viruses are important factors for their efficient replication in CNS cells. Thus, the zoonotic H5N1 virus replicates more efficiently in all the CNS cells, an event that was a consequence of efficient viral attachment and infection (Siegers et al., 2019). Nevertheless, viruses are usually isolated in low titers and are poorly identified by immunohistochemistry, indicating that they reach the CNS, but probably replicate inefficiently at this site. Interestingly, Short et al. (2017) showed that in the absence of effective viral replication for H1N1 or H5N1, an increase of pro-inflammatory cytokines such as IL-6, IL-8, and TNF-α are induced in the respiratory tract and CNS, to modulate neuroinflammatory events. Considering the existence of KLK1, KLK5, and KLK12 in the nervous system, their role as proteases in IAV infection, it would be interesting to determine whether they contribute to influenza viruses entrance and replication in CNS cells. It has been shown in three viral strains that hemagglutinin subtypes are cleaved by a wide range of proteases, including trypsin, matriptase, human airway tryptase, plasmin, and the aforementioned KLK5 and KLK12 (Straus and Whittaker, 2017), suggesting that a broad spectrum of proteases may contribute to the pathogenicity of H1N1, H2N2, and H3N2 strains.

Some reports connect CNS viral infections with the development or diagnosis of various CNS disorders. For example, detection of human herpesvirus 8 (HHV-8) in postmortem brains has led some scientists to propose that HHV-8 has a neuroinvasive and neuropersistent potential (Chan et al., 2000). Interestingly, it has been shown that infection by this virus in the prostate produces an increase in serum levels of KLK3 (Henning et al., 2017).

As we mentioned before numerous studies have reported an interaction between viral infection and neural cells leading to multiple sclerosis. Marashi et al. (2018) examined peripheral blood looking for DNA sequences of HHV-8 in 54 multiple sclerosis patients and 130 healthy subjects, detecting the virus in 18.5% of the patients and 3% of controls. HHV-8 infection has also been reported in patients with acquired immune deficiency syndrome (AIDS) associated with Kaposi sarcoma (Corbellino et al., 1996; Ogoina et al., 2011). An association between HHV-8 and neurological disorders is highly likely, whether HHV-8 infection alters the expression of KLK3 or other KLKs when reaching the CNS, remains unclear.

Viruses that cause chronic infection have also been related to neuroinflammatory states; among them, the acquired immunodeficiency virus type 1 (HIV-1) and the hepatitis B virus (HBV; Aygören-Pürsün et al., 2016; Sun et al., 2017) may have some unexplored relation with KLKs. HIV-infection in the CNS is a significant step of systemic infection that can be detected shortly after primary exposure and initial viremia (Davis et al., 2012). HIV-related neurological disease is characterized by acute inflammation and neuronal damage, leading to encephalitis (Valcour et al., 2012). In a neuroinflammatory process, Boven et al. (2003) reported high expression of PAR-1 and pro-thrombin at the protein and mRNA levels, respectively. Intense immunoreactivity was observed in human brain astrocytes of patients suffering from HIV encephalitis, suggesting that its high expression might contribute to brain inflammation and neuronal damage during HIV-1 infection. KLK6 participates in astrogliosis by activating PAR-1 in astrocytes (Scarisbrick et al., 2012b), and an elevated PAR-2 expression has been described in neurons of HIV-1 patients associated to dementia, and its activation seems to prevent neuronal cell death (Noorbakhsh et al., 2005). However, on this date, the possible participation of KLKs in these infectious conditions has not yet been explored.

Considering the ability of KLKs to degrade ECM components, activate signaling pathways related to cell survival and immune response, in addition to its high expression in the CNS, it would be interesting to explore the relationships that may exist between protease function and neurotropic pathogens, which can reach and infect the CNS. In this context, Theiler’s murine encephalomyelitis virus (TMEV) is commonly used as a model of multiple sclerosis due to its ability to infect neurons and cause demyelination and axonal damage (Tsunoda and Fujinami, 2010). TMEV model has been used in studies involving Klk6 and the neuropathological mechanisms associated with this disorder (Blaber et al., 2002; Scarisbrick et al., 2002, 2012b; Spencer et al., 2015). Additionally, other kallikrein family members, such as Klk1, Klk7, Klk8, and Klk10 are expressed in the brain and spinal cord of mice infected with TMEV (Panos et al., 2014).

Neurotropic viruses may negatively impact the normal physiological activity of the CNS by affecting the integrity of the cerebral tissue due to cellular destruction as a result of lytic infection; thus, they can be considered risk factors for developing an alternate neuroimmune response and neurodegenerative diseases such as Parkinson’s and Alzheimer diseases (Ringheim and Conant, 2004; Itzhaki, 2018; Olsen et al., 2018). Further, a chronic immune activation may lead to neuroinflammation and subsequently to neurodegeneration. Considering that almost all KLKs are expressed to some extent in the CNS, these data emphasize the need for a more detailed characterization of the effects produced by viral infections on the routes leading to neurodegeneration and KLKs activation. Projects on the specific proteins that participate in the life cycle of viruses, particularly those involved in attachment to the host cell, may determine the viral tropism and explain why viruses can cross the BBB and cause severe or fatal diseases in humans. Thus, novel ideas on the role of KLKs and other proteases during different steps of viral infection are needed.

## Conclusions

The evidence presented above demonstrates that an appropriate balance between expression levels and activity of proteases is crucial for the homeostasis of CNS. The dysregulation of certain KLKs, that is, KLK1 and KLK7, but mainly KLK6 and KLK8, may contribute to the development of neurodegenerative or neurological disorders such as Alzheimer’s and Parkinson’s diseases, memory disorders or mental illness. If this dysregulation is secondary to neurotropic virus infection appears as an important question to answer that may develop a new field of scientific research, especially now that some viruses have been linked to the development of neurodegenerative diseases, having a major role in the pathogenesis of the disease. This opens the discussion about the impact of KLKs dysregulation during viral infections affecting the CNS and how KLKs may affect the viral replicative cycle. Other open questions are the role of KLKs in the bioavailability of growth factors important for CNS homeostases such as BDNF or NGF, and their role in the modulation of the inflammasome, among others. This evidence points out the importance to study certain KLKs, which are overexpressed in the CNS as key targets for diseases that have affected human well-being for centuries.

## Author Contributions

CM and PE researched and wrote this review. CM, PE, and CO conceived the research, and CF assisted in writing and editing.

## Conflict of Interest

The authors declare that the research was conducted in the absence of any commercial or financial relationships that could be construed as a potential conflict of interest.
